# Voxel-wise comparisons of cellular microstructure and diffusion-MRI in mouse hippocampus using 3D Bridging of Optically-clear histology with Neuroimaging Data (3D-BOND)

**DOI:** 10.1038/s41598-018-22295-9

**Published:** 2018-03-05

**Authors:** H. B. Stolp, G. Ball, P.-W. So, J.-D. Tournier, M. Jones, C. Thornton, A. D. Edwards

**Affiliations:** 1 Centre for the Developing Brain, School of Biomedical Engineering and Imaging Sciences, St Thomas’ Hospital, King’s College London, London, SE1 7EH United Kingdom; 2 Department of Neuroimaging, Maurice Wohl Clinical Neuroscience Institute, Institute of Psychiatry, Psychology and Neuroscience, King’s College London, London, SE5 9NU United Kingdom; 3 Developmental Imaging, Clinical Sciences, Murdoch Children’s Research Institute, Melbourne, 3052 Australia; 4 Present Address: Department of Comparative Biomedical Science, Royal Veterinary College, London, NW1 0TU United Kingdom

## Abstract

A key challenge in medical imaging is determining a precise correspondence between image properties and tissue microstructure. This comparison is hindered by disparate scales and resolutions between medical imaging and histology. We present a new technique, 3D Bridging of Optically-clear histology with Neuroimaging Data (3D-BOND), for registering medical images with 3D histology to overcome these limitations. *Ex vivo* 120 × 120 × 200 μm resolution diffusion-MRI (dMRI) data was acquired at 7 T from adult C57Bl/6 mouse hippocampus. Tissue was then optically cleared using CLARITY and stained with cellular markers and confocal microscopy used to produce high-resolution images of the 3D-tissue microstructure. For each sample, a dense array of hippocampal landmarks was used to drive registration between upsampled dMRI data and the corresponding confocal images. The cell population in each MRI voxel was determined within hippocampal subregions and compared to MRI-derived metrics. 3D-BOND provided robust voxel-wise, cellular correlates of dMRI data. CA1 pyramidal and dentate gyrus granular layers had significantly different mean diffusivity (p > 0.001), which was related to microstructural features. Overall, mean and radial diffusivity correlated with cell and axon density and fractional anisotropy with astrocyte density, while apparent fibre density correlated negatively with axon density. Astrocytes, axons and blood vessels correlated to tensor orientation.

## Introduction

Medical imaging technologies, such as position emission tomography (PET), computed tomography (CT) and magnetic resonance imaging (MRI), have facilitated substantial advances in the diagnosis, monitoring and treatment of disease. A key challenge in medical imaging is to understand how the image properties correspond to specific elements of the tissue microstructure. The side-by-side comparison with histologically stained tissue samples can serve as a validation for medical imaging. However, this comparison is challenged by substantial differences in scale and resolution between the two modalities. Imaging data is typically produced from 1–2 mm^3^ voxels in patients, compared to high-resolution (1–10 μm/pixel) but two-dimensional microscopy data, resulting in inherent limitations and inaccuracies in comparisons.

Diffusion MRI (dMRI) has proven sensitive to microstructural changes in neuropathological disease, showing capacity to distinguish between phases of disease (e.g. in Alzheimer’s disease, Amyotrophic Lateral Sclerosis, Epilepsy^1–3^), and to identify changes associated with neuropathology. For example, increased mean diffusivity and decreased fractional anisotropy are consistent findings in Alzheimer’s disease and are sufficiently sensitive to distinguish the prodromal form from age-matched healthy controls^4^, potentially detecting early cytoarchitectural changes as a result of neurofibrillary tangle formation^5^. While dMRI is extremely sensitive to microscopic changes that occur as a result of pathological (i.e. demyelination, axonal loss, oedema, inflammation), as well as developmental (e.g. dendritic arborisation, axonal growth) processes, assigning changes in dMRI metrics to specific microstructural alterations is an ill-posed problem with multiple possible solutions. This complexity is highlighted when considering changes in diffusion-based metrics such as fractional anisotropy which may be due to changes in myelin, membrane permeability, axonal number or size, or a combination of all of those factors^6^. This has led to a number of recent investigations into the histological correlates of the diffusion signal^7,8^.

Traditional histological techniques require the sectioning of tissue samples followed by digital reconstruction and an in-plane comparison with co-aligned imaging data. This process does not account for the true 3D structure of the intact tissue sample and assumes precise through-plane alignment of both modalities. Resolution and contrast differences also limit structure recognition and therefore subsequent alignment of the disparate datasets. In the complex environment of injury, particularly in the developing or ageing brain, where multiple cellular changes are occurring together, a new method for histological assessment and interrogation of medical imaging is required.

The recent development of tissue clearing techniques has made it possible for a true comparison of 3D tissue microstructure with 3D medical imaging. These clearing methods work by masking^9–12^ or removing^13,14^ the light-refracting lipids within the tissue, allowing detection of cellularly-located fluorescent proteins over large tissue volumes. These tissue preparation methods also take advantage of technical advances in light microscopy such as specialised objectives with long working distance and high numerical aperture, and altered light paths to minimise noise from out-of-focus tissue and speed up image acquisition^11,15,16^. Furthermore, a number of these clearing procedures are compatible with immunohistochemistry^13,17,18^, enabling the simultaneous assessment of multiple cellular populations.

Here we present an analysis of dMRI metrics and cellular microstructure, using 3D-BOND (3D Bridging of Optically-clear histology with Neuroimaging Data), a technique for registering medical images with 3D histology. This analysis has been performed in the hippocampus, a brain region with a complex microstructure and a primary focus of many disease-related MRI studies. Bridging the gap between low-resolution MRI and cellular-resolution histology, and allowing cell-specific, 3D analysis of the histological data influencing MRI, is paramount to our understanding and accurate clinical translation of *in vivo* MRI to neurodevelopment and neuropathology.

## Materials and Methods

**Figure 1:**
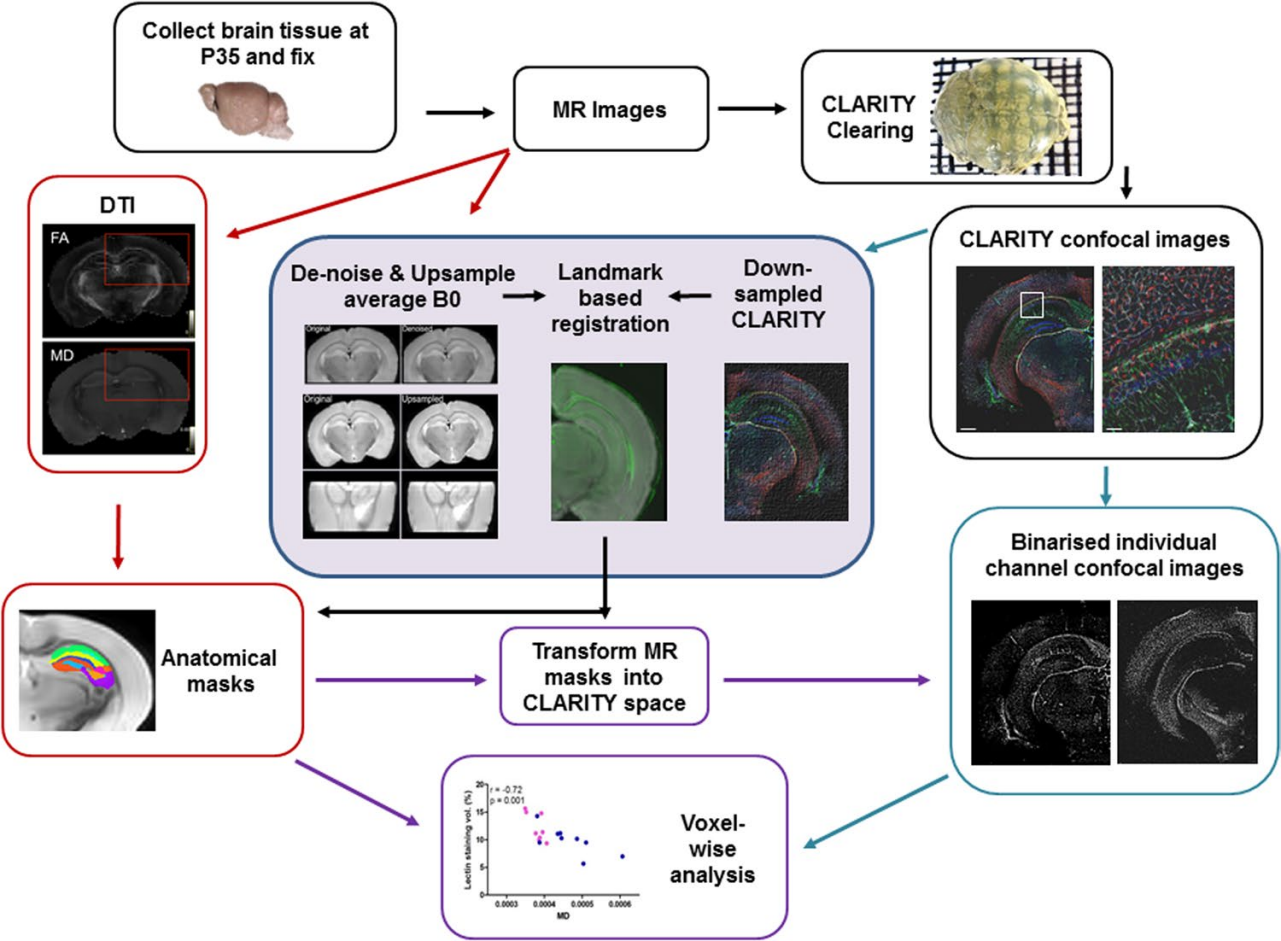
3D-BOND Workflow. *Ex vivo*, high-resolution dMRI was performed on fixed mouse brains which were then optically-cleared with CLARITY-based processing. Tissue was fluorescently stained with CLARITY-validated, cell-specific antibodies and imaged using confocal microscopy. MR and confocal images were processed and registered before voxel-wise analyses.

Brain tissue was prepared for MRI, CLARITY-based histological processing, registration and analysis as indicated below, and in the schematic diagram in Fig. 1.

### Tissue preparation

All animals were housed in standard conditions, with a 12 h light/dark cycle in individually ventilated cages, and treated in accordance with the UK Animals (Scientific Procedures) Act 1986 and local King’s College London Animal Welfare and Ethical Review Body guidelines under project licence 70/8367. Adult C57Bl/6 mice (n = 9) were killed by cervical dislocation; brain tissue was dissected out, cut to a 4–6 mm block from approximately Bregma 1.0 mm to −4.5 mm, and fixed by immersion in 4% paraformaldehyde (PFA) for 48 hours. Tissue was then washed in phosphate-buffered saline (PBS, pH 7.4) for at least 7 days prior to MRI.

### *Ex vivo* MRI

Fixed brain tissue blocks were held in a customised sample holder and immersed in Fomblin (Galden SV40, Performance Fluids, UK) for MRI on a 7*T* horizontal bore MRI scanner (Agilent Technologies Inc, Walnut Creek, CA, USA) using a quadrature volume radiofrequency coil (26 mm inner diameter; Rapid Biomedical, Rimpar, Germany), in a temperature controlled environment (21 °C). Diffusion MR images were obtained using a fast spin echo (FSE) sequence with the application of diffusion gradients in 42 directions at a b-value of 1500 s/mm^2^, repetition time (TR) = 3500 ms, echo time (TE) = 41.30 ms, Δ = 26 ms, δ = 5 ms, 40 averages, 22.5 hour scan time. Coronal contiguous slices (21) of 0.2 mm thickness were collected using the smallest field of view possible for the specific tissue sample and the matrix was adjusted to produce a final voxel size of 125 × 125 × 200 µm.

The 42-direction diffusion data was initially denoised using a local Principal Component Analysis (PCA) filter^19^. Diffusion tensors were calculated with a weighted least-squares fit at each voxel and fractional anisotropy (FA), mean diffusivity (MD), radial diffusivity (RD) and parallel diffusivity (PD) maps derived using FSL’s *dtifit*^20^. Apparent fibre density (AFD) maps were derived using MRTrix3 (http://www.mrtrix.org).

### Histological tissue processing

Following MR imaging, tissue blocks were cut into 2 mm coronal slices and processed with the passive CLARITY protocol^13,14,16^; this slow, passive clearing method overcomes the issue of tissue inflation assocated with the original method^21^. Briefly, tissue was immersed in hydrogel (4% acrylamide, 0.05% bis-acrylamide, 4% PFA in PBS) at 4 °C for 48 hours. Samples were deoxygenated with nitrogen using a Schlenk line attached to a vacuum flask before polymerisation of the hydrogel at 37 °C. Tissue samples were then transferred to clearing buffer (4% sodium dodecyl sulphate (SDS), 200 mM Boric acid, pH 8.5) at 37 °C until clear (2–3 weeks).

Cleared tissue was washed in sodium borate buffer (SBB, 1 M boric acid, pH 8.5, 48 h) before incubation in primary antibodies diluted in SBB with 1% Triton-X100 (see Table 1 in supplementary material). Tissue was incubated in antibodies for 7–10 days, with 24 hour SBB washes between primary and secondary incubations. Secondary antibodies raised in goat or donkey against mouse, rabbit, goat, chicken or guinea pig were used, attached to either 488 nm, 546 nm or 647 nm fluorophores (AlexaFluor, Life Technologies, diluted 1:500 in SBB). Prior to imaging, tissue was incubated in 4′,6-diamidino-2-phenylindole (DAPI, 1:1000 in SBB), washed in SBB and placed in a refractive index matched solution (RIM^14^) for 24 hours.

**Figure 2:**
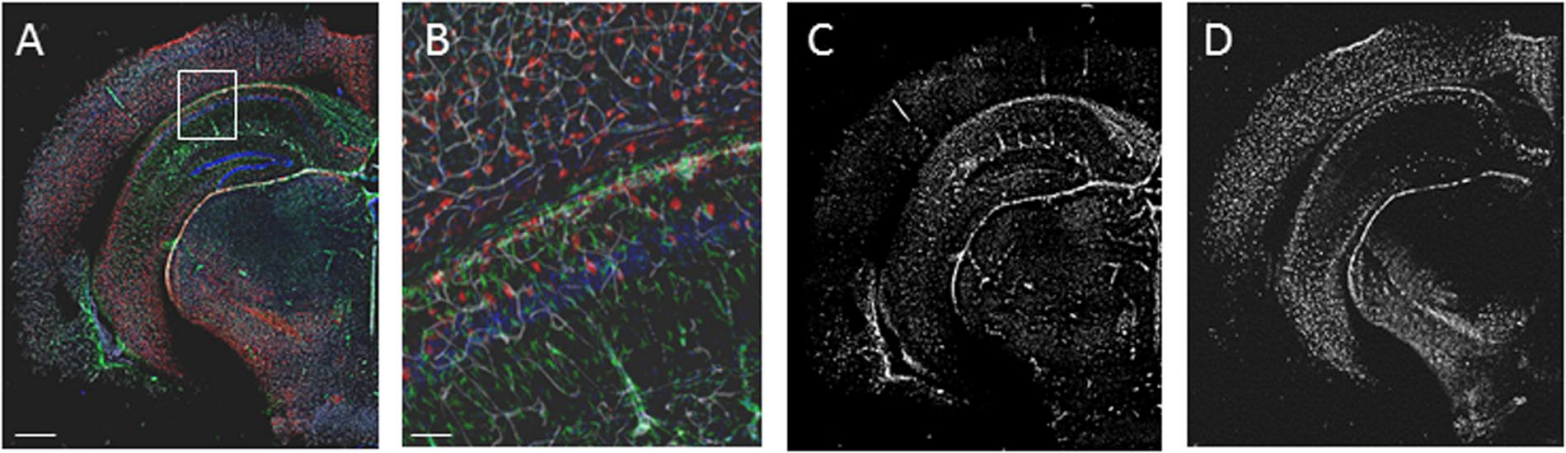
3D histological data from optically-clear tissue at macro- and microscale. CLARITY-processing combined with immunohistochemistry and confocal microscopy allowed whole brain imaging at cellular resolution. (**A**) GFAP-positive astrocytes (green), parvalbumin-positive interneurons (red) and tomato lectin-positive blood vessels (white) with DAPI stained cell nuclei (blue) in a cerebral hemisphere from an adult mouse brain. (**B**) High magnification image of boxed region from (**A**) shows the resolution of the image. (**C** and **D**) Individual channels were separated to show regional differences in cellular distribution, (**C**) green channel - GFAP positive astrocytes are particularly dense within the hippocampus and white matter, (**D**) red channel - parvalbumin positive interneurons are predominantly found within the cortex and pyramidal layers of the hippocampaus. Scale bar: **A**,**C** and **D** = 1 mm, **B** = 100 μm.

Confocal imaging was performed on an Eclipse Ni-E Upright microscope with a Plan Apo 4× objective (numerical aperture [NA] 0.2, working distance [WD] 10 mm), a Plan Fluor DIC L N1 10× objective (NA 0.30, WD 4 mm) or Plan Apo VC DIC N2 20× objective (NA 0.75, WD 1 mm) using NIS Elements C software (Nikon). Images of whole samples were produced with multi-frame tiling (512 × 512 pixels/frame, with 15% overlap for stitching), using unidirectional scanning at 405 nm, 488 nm, 561 nm and 642 nm laser wavelengths. Laser intensity was set at four points throughout the tissue depth, and was automatically interpolated between each point, to ensure the full intensity range was used throughout the z-stack. Images were converted to tiff format for registration and analysis. Fully compiled datasets show the histological microstructure of the brain for four specific cellular subtypes in a single sample, at an in-plane resolution of 2.4 μm × 2.4 μm (Fig. 2A,B), which can be projected in 3D to show the detailed microstructure of the hippocampus (Suppl. Figure 1).

### Image Processing

Confocal images were aligned with the Registration plug-in for ImageJ using rigid registration to correct any slice-wise alignment errors during acquisition. Separate colour channels were individually processed in ImageJ to normalise contrast across the image stack (40 × 40 pixel block radius, standard deviation of 3) and enhance contrast-to-noise (CNR, gamma correction 2.0; examples shown in Fig. 2C,D). Manual analysis of regions of interest in multiple datasets were performed to confirm that there was no effect of contrast enhancement on cell populations being analysed. Data from confocal image sets was only included where staining showed consistent distribution across a brain region and cell morphology, as well as a CNR > 4 to facilitate automated analysis. Images were made binary by applying a threshold at 2× noise, again validated by manual and automated analysis over multiple thresholds and tissue regions.

### Registration

To form a source volume for registration, the six B0 volumes were averaged, denoised using a 3D non-local means (PRINLM) filter and upsampled by a factor of 5^22^ (Suppl. Figure 2). This upsampling factor represents a midpoint between the two modalities, which was within the capacity limit of the upsampling method. Both denoising and upsampling processes take advantage of the pattern redundancy in image data, allowing a patch-based reconstruction that outperforms traditional interpolation^23^. As a corresponding target image for registration, confocal images were downsampled in-plane, using standard linear interpolation in Matlab (The Mathworks, Inc; Natick, MA), to match as closely as possible to the final resolution of the upsampled average B0 volume (25 × 25 µm).

To achieve spatial correspondence, the upsampled average B0 volume and downsampled 3D confocal image stack were aligned using a dense landmark-based registration scheme. Landmark identification was facilitated by the distinct 3D structure of the hippocampus (see Suppl. Figure 3) and 58 distinguishable coordinates from the left and right hemispheres at multiple rostro-caudal positions (approximately equating to Bregma positions: −1.1 mm, −1.3 mm, −1.7 mm, −2.1 mm and −2.7 mm) were included in the registration schema (based on the atlas of Paxinos & Franklin, 2012; and the online mouse brain atlas http://www.mbl.org/atlas170/atlas170_frame.html; Suppl. Table 1). For each MRI data set, multiple 2 mm slices from the brain sample were registered, utilising the majority of landmarks available. However, due to variation in contrast and sampling between MR and confocal images not all 58 positions were available for registration in each paired data set; only those landmarks which could be accurately identified were used, from a minimum of three rostro-caudal positions. Across all samples, the average number of landmarks used was 33 ± 4 (±S.D; range 30–37). Once annotated, the MR volume was registered to the confocal stack using affine registration, minimising the distance between landmarks (root mean squared distance, RMS, example shown in Suppl. Figure 4A). The RMS was then used as a measure of intra-sample registration error, with an average RMS distance between corresponding landmarks of 0.168 ± 0.031 mm (Supp. Table 2). These values were driven by slightly greater registration error at the lateral boarders of the hippocampus compared to the midline, therefore regions of interest (ROIs) for further analysis (described below) were selected from medial hippocampal regions to further minimise any potential confounding effect of lateral registration error. RMS was also used to assess intra-investigator variation, assessed by placing landmarks five times in the same MR-confocal dataset, with a test-retest reliability of 0.9 (Cronbach’s alpha).

### Analysis

To standardise voxel selection for analysis, hippocampal masks from the Australian Mouse Brain Mapping Consortium (AMBMC, www.imaging.org.au/AMBMC) for the primary Cornu Ammonis (CA)1 pyramidal layer and stratum radiatum (CA1sp and CA1sr, respectively), Dentate Gyrus (DG) granular and molecular layers (DGgl and DGml, respectively) were registered to each sample’s upsampled average B0 volume using an intensity-based affine and nonlinear registration based on b-spline deformation (IRTK)^24^. Hippocampal labels were simultaneously propagated via the average B0 onto the corresponding confocal image stacks, and the corresponding fractional anisotropy (example shown in Suppl. Figure 4B) and mean diffusivity maps, at their original resolutions.

Voxel-wise analysis of histological data was performed in ImageJ. For each voxel in the hippocampal ROIs, binarised data from each colour channel were separated and the area of staining for each confocal slice calculated using the Measure Tool. The area of staining was calculated as a percentage of the total 3-dimensional volume defined by each voxel and compared to the MRI-derived metrics from the corresponding voxel in the original dMRI space for each of the selected hippocampal sub-regions.

The CLARITY images were processed to generate the corresponding 3D structure tensor maps, consisting of the matrix of second-order spatial derivatives after Gaussian smoothing^25^. A smoothing factor of 5 μm was used to detect microstructure within each cell-specific light microscopy dataset. Within each imaging voxel, the dominant orientation was estimated as the minor eigenvector of the structure tensor. Each of these orientations was then expressed as the spherical harmonic representation of a delta function pointing along the corresponding orientation. Finally, fibre orientation distributions were computed within larger voxels by summing the corresponding spherical harmonic coefficients over all voxels (at the original CLARITY resolution) contained within each voxel at the target MRI resolution. These were then displayed as orientation glyphs using the MRView application (included as part of *MRtrix3*).

### Statistics

Of the 9 brains samples used in this study, 6 were used to optimise the 3D-BOND pipeline, while 3 were processed through the entire pipeline. All data are presented as mean ± SEM unless otherwise stated. Grouped data were compared using a one-way ANOVA. Correlations between each diffusion metrics and the 6 different types of cellular data were performed using Prism (GraphPad). Statistical significance was set at p < 0.05, and a false discovery rate (FDR) correction was performed using the Benjamini-Hochberg procedure to correct for comparisons of multiple diffusion metrics.

## Results

### Diffusion characteristics of the hippocampus

**Figure 3:**
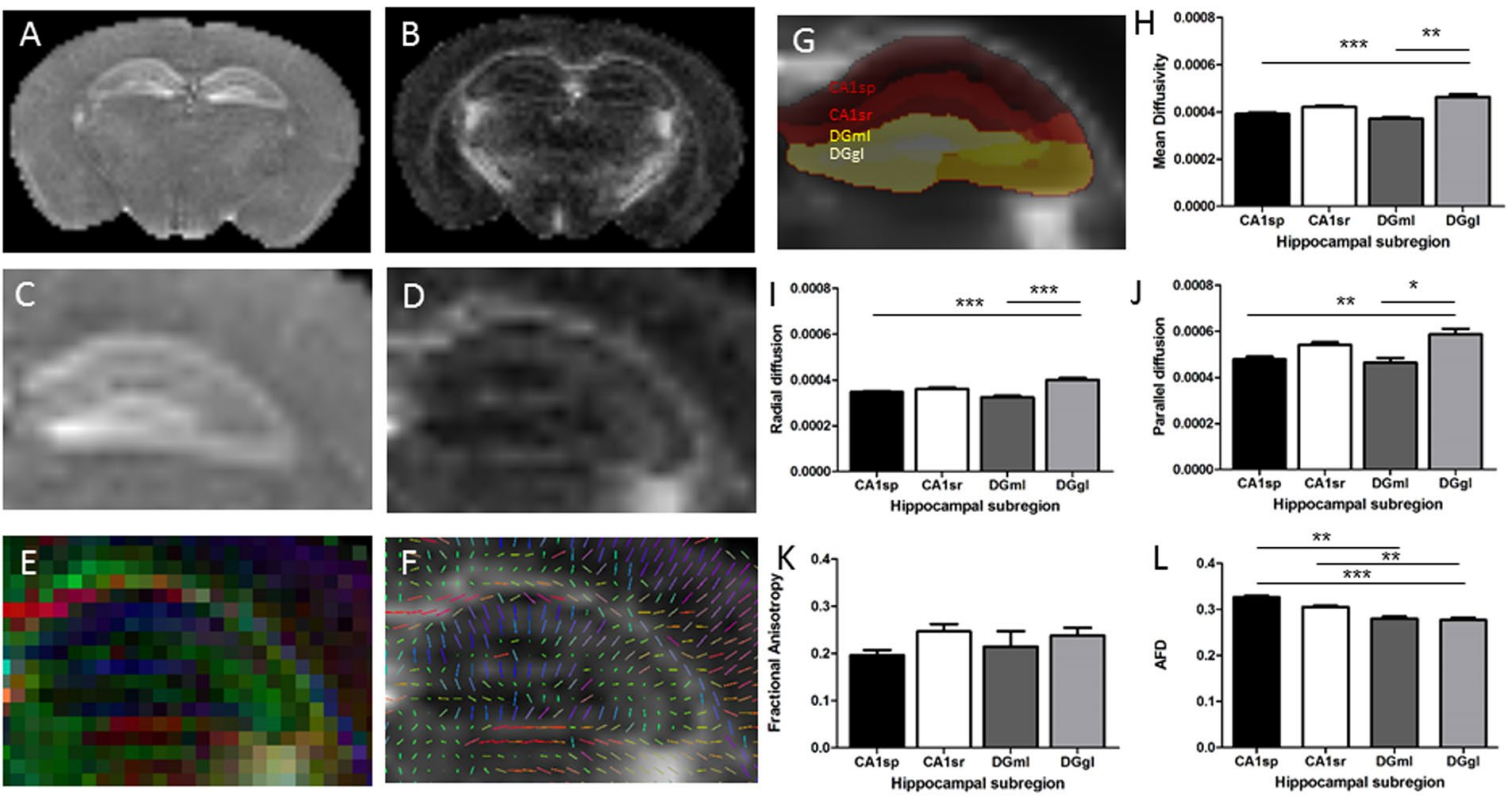
MRI imaging & processing. Mean diffusivity (MD; **A**,**C**) and fractional anisotropy (FA; **B**,**D**) maps were calculated from 42-direction dMRI. Data was also visualised as RGB and line vector maps of diffusion directions (**E**,**F**). Diffusion metrics were calculated for two of the CA1 hippocampal layers (CA1sp and CA1sr) and two of the DG hippocampal layers (DGgl and DGml), illustrated in G. Variations in mean diffusivity (**H**), radial diffusion (**I**) parallel diffusion (**J**), fractional anisotropy (**K**), and apparent fibre density (**L**) were assessed, showing significant differences between the DGgl and other layers. *p < 0.05, **p < 0.01, ***p < 0.001.

The macrostructure of the hippocampus is alternate layers of cell dense and cell sparse regions, with complex layer-specific patterns of axon projections, dendritic arborisation, vascular plexi and glia. High resolution *ex vivo* dMRI was performed to assess the diffusion characteristics of these hippocampal subregions (Fig. 3A–F), with a focus on the CA1 pyramidal layer (CA1sp) and dentate gyrus granular layer (DGgl) as two cell dense regions, and the CA1 stratum radiatum (CA1sr) and dentate gyrus molecular layer (DGml) as neighbouring cell sparse, projection heavy layers. Layering within the hippocampus could be seen on maps of mean diffusivity (Fig. 3A,C) and fractional anisotropy (Fig. 3B,D). Specific hippocampal layers were identified using the Australian Mouse Brain Mapping Consortium atlas (AMBMC, www.imaging.org.au/AMBMC, Fig. 3G) and diffusion values within these layers were calculated and compared.

A significant difference in diffusion was seen between layers with the diffusion tensor imaging (DTI) metrics of mean diffusivity, radial diffusivity and parallel diffusivity. Mean diffusivity was highest in the DGgl (0.46 ± 0.012 × 10^−3^ mm^2^.s^−1^), significantly different from the CA1sp (0.39 ± 0.006 × 10^−3^ mm^2^.s^−1^, p < 0.0001) and DGml (0.42 ± 0.006 × 10^−3^ mm^2^.s^−1^, p < 0.01, Fig. 3H). Radial and parallel diffusion values were slightly different, but showed the same pattern and statistical significance (Fig. 3I,J). No statistically significant difference in fractional anisotropy was observed across the hippocampal layers with the current imaging paradigm (Fig. 3K), though fractional anisotropy was also generally higher in the DGgl compared to the CA1sp (DGgl 0.238 ± 0.016, CA1sp 0.196 ± 0.011). This data supports previous reports indicating the sensitivity of mean diffusivity (and other diffusivity measures) to local changes in microstructure. Mean apparent fibre density (AFD) was also measured as an alternative metric for assessing tissue microstructure to those obtained using DTI (Fig. 3L). This measure showed the highest mean AFD value in the CA1sp region of the hippocampus (0.32 ± 0.001) which was significantly higher than both the DGgl (0.28 ± 0.06, p < 0.001) and DGml (0.28 ± 0.05, p < 0.01). The mean AFD in the CA1sr (0.31 ± 0.003) was also significantly higher than in the DGgl (p < 0.01).

### Correlation between diffusion and cellular microstructure of the hippocampus

**Figure 4:**
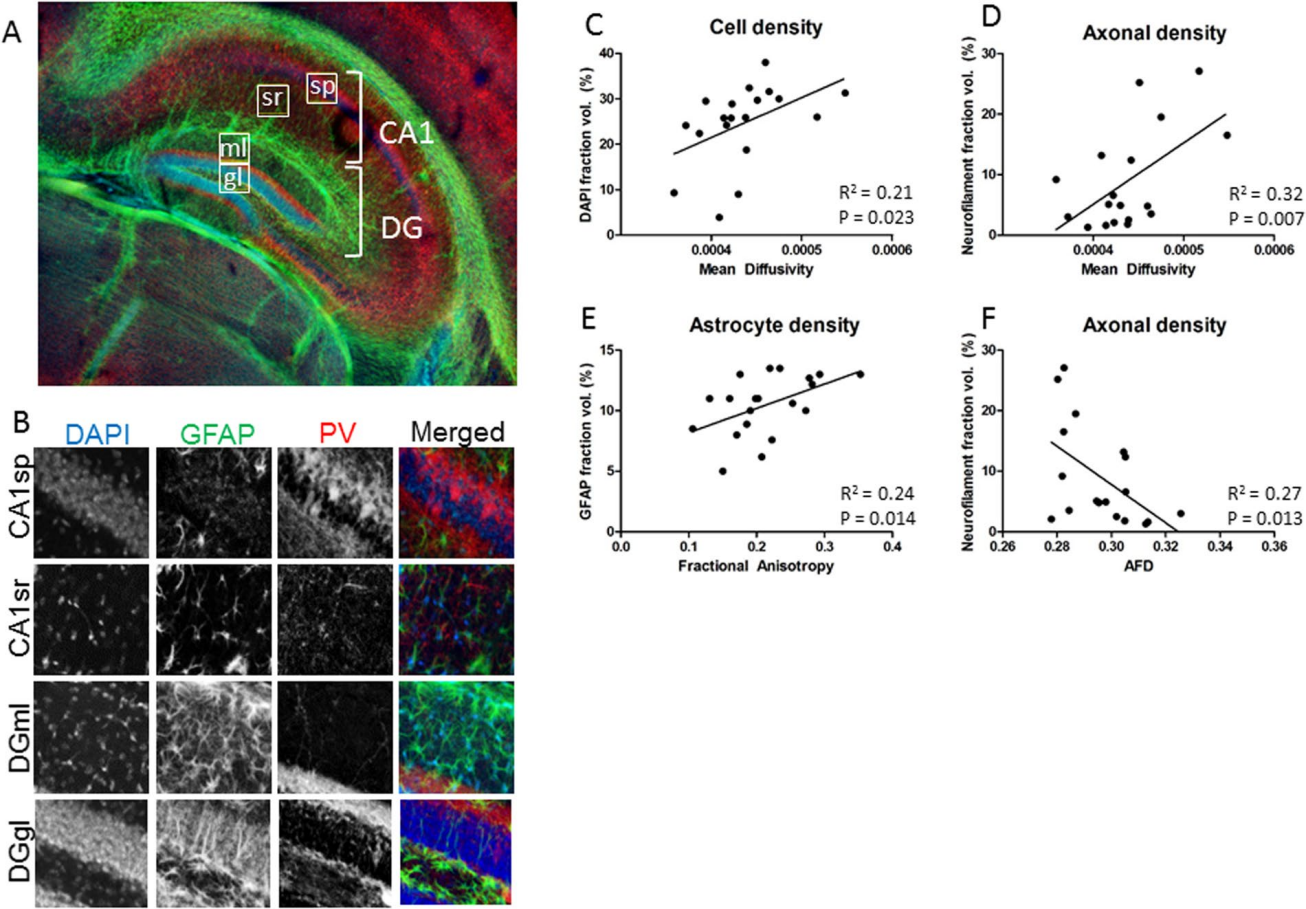
Differences in diffusion in hippocampal subregions. (**A**,**B**) Cellular imaging of the hippocampus shows regional variation in microstructure. Example voxels from each hippocampal region are outlined in (**A**), and showed at higher magnification in (**B**). DAPI (cell nuclei), PV (interneuron population) and GFAP (astrocytes) are differentially distributed within the CA1sp, CA1sr, DGgl and DGml cell layers. (**C**) Mean diffusivity correlates with DAPI staining area, reflective of cell density. (**D**) Mean diffusivity also correlates with axonal density. (**E**) Fractional anisotropy only correlates with astrocytes staining density, as shown with GFAP. (**F**) Axonal density, quantified from neurofilament staining area, negatively correlated with apparent fibre density in the hippocampal subregions examined.

While CLARITY has been shown to be compatible with immunohistochemistry^13^, only a very limited number of antibodies have been validated. We therefore established a comprehensive collection of neurodevelopmental and neuropathological cellular markers assessed in CLARITY-processed tissue (for details and examples see Suppl. Figure 5 and Suppl Table 3). For the implementation of the 3D-BOND pipeline, we focused on six well characterised antibodies or stains that defined distinct features of the hippocampal microstructure (see Fig. 4A,B). DAPI staining of cell nuclei was used to assess cell density, neurofilament staining to show axons, and a subclass of large, arborized interneurons detected with antibodies against parvalbumin. GFAP (glial fibrillary acid protein) and Iba1 (ionized calcium binding adaptor molecule) were used as markers of astrocytes and microglia respectively and tomato lectin to show the vasculature (arterioles, venules and capillaries).

We correlated the density of staining of each microstructural element with the corresponding diffusion metric of each voxel within each hippocampal ROI (Table 1, Fig. 4C–F). Cell density showed a weak positive correlation with mean diffusivity (Fig. 4C), as well as with radial and parallel diffusivity (r^2^ = 0.21, 0.23 and 0.15 respectively), though this was only statistically significant following post-hoc Bonferroni correction for mean and radial diffusivity (p = 0.023 and 0.018 respectively). There was no correlation between fractional anisotropy or mean apparent fibre density and cell density. Statistically significant positive correlations were also found between axon density within a voxel and mean, radial and parallel diffusivity (r^2^ = 0.32, p = 0.007; r^2^ = 0.40, p = 0.003; and r^2^ = 0.18, p = 0.038, example in Fig. 4D) as well as a negative correlation between axon density and mean apparent fibre density (r^2^ = 0.27, p = 0.013, Fig. 4F). As with cell density, there was no correlation between axonal density and fractional anisotropy. There was, however, a statistically significant positive correlation between fractional anisotropy and astrocyte density within hippocampal voxels (r^2^ = 0.24, p = 0.014, Fig. 4E). There was also a weak correlation with astrocyte density and parallel diffusion and with mean AFD (r^2^ = 0.18 and r^2^ = 0.17; not significant following FDR correction), but not for mean or radial diffusivity. The density of parvalbumin interneurons did not correlate significantly with any measures on a voxel-by-voxel basis across the hippocampus. There was also no correlation between resting Iba1-positive microglia and diffusion metrics at a whole tissue level (Table 1). There was a weak negative correlation between blood vessel density and all diffusion metrics examined in this study, though this was not significant for any measure following FDR correction.

**Table 1:** Goodness-of-fit between DTI metrics and voxel cellular content in the hippocampus.

	FA	AFD	MD	RD	PD
Cell density	0.01	*0*.02	0.21*	0.23*	0.15
Axons	*0*.*04*	*0*.27*	0.32*	0.40*	0.18*
Parvalbumin interneurons	0.01	*0*.*05*	0.04	0.03	0.04
Astrocytes	0.24*	*0*.*17*	0.09	0.01	0.18
Microglia	*0*.*01*	*0*.*01*	*0*.*12*	*0*.*12*	*0*.*09*
Blood vessels	*0*.*16*	0.13	*0*.*17*	*0*.*08*	*0*.*21*

Note: Values presented are R^2^, negative correlations are indicated in italics. * represents statistically significant correlation between features (p < 0.05 following FDR correction using Benjamini_hochberg procedure). Data included from voxels within the CA1sp, CA1s, DGgl and DGml layers of the hippocampus.

### Local correlations between microstructure and diffusion

**Figure 5:**
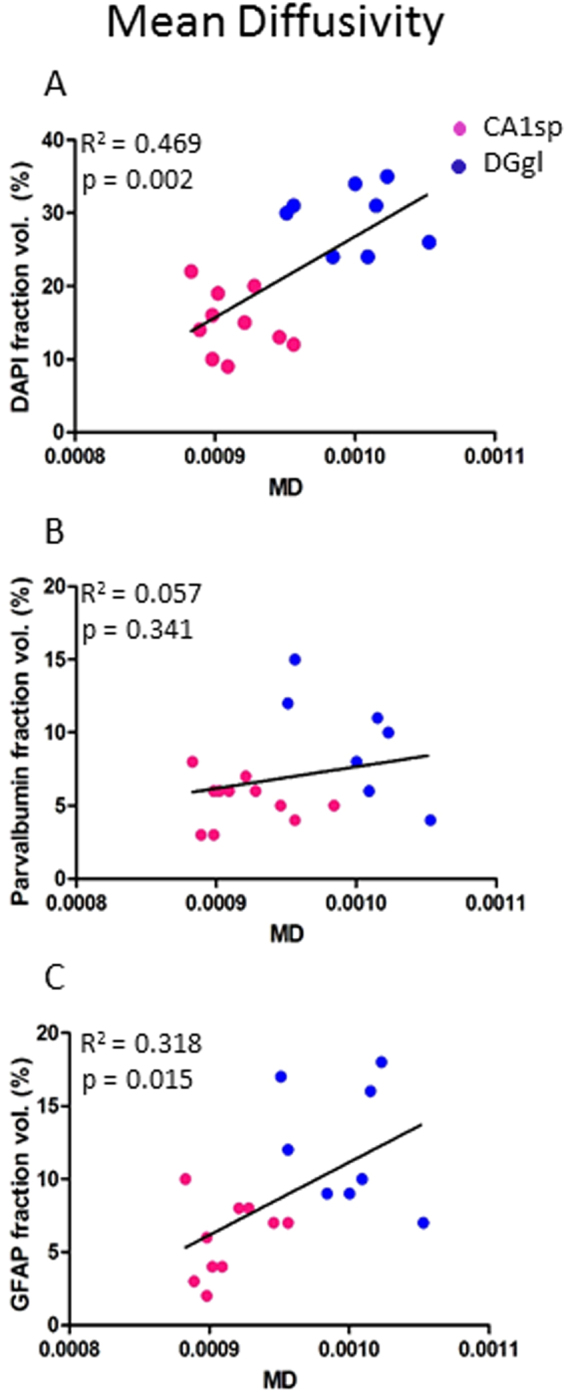
Secondary influences of microstructure on diffusion metrics in hippocampal subregions. When the two cell dense regions were examined for the way their cellular microstructure correlated with mean diffusivity, there was clear separation of data from the CA1sp region (pink dots) and the DGgl region (blue dots). This resulted in a statistically significant correlation between mean diffusivity and cell density (**A**) and astrocyte density (**C**), but not parvalbumin interneuron density (**B**).

Given the finding of a correlation between cell density and mean diffusivity, the CA1sp and DGgl were further analysed for secondary associations between cell composition and diffusivity. A clear separation between cell densities in the two layers could still be seen, with the correlation between mean diffusion and cell density remaining (Fig. 5A). Parvalbumin interneuron density and GFAP positive astrocytes also showed distinct patterns between these regions, though in the case of the interneurons this grouping did not correlate significantly with mean diffusivity. There was a clear difference in the area of the CA1sp and DGgl that contained GFAP positive astrocytes (Fig. 5C), and this correlated significantly with mean diffusivity (r^2^ = 0.32, p = 0.015).

### Structure tensor analysis of histological tissue, in comparison with dMRI tensors

**Figure 6:**
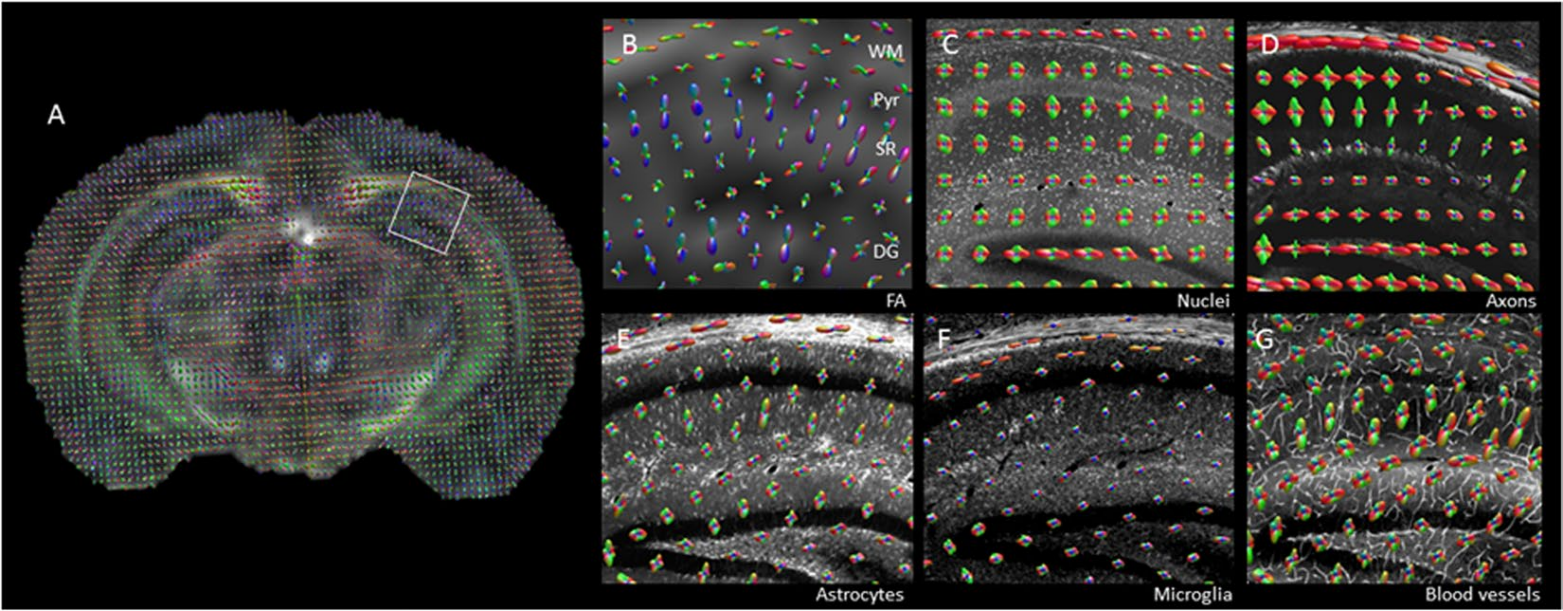
Directionality of microstructure aligns with dMRI. (**A**) Orientation density functions (ODFs) were calculated from dMRI data, and were overlaid on the fractional anisotropy image of the brain. dMRI data was aligned with 3D-histology from CLARITY processed tissue. ODFs were compared in the hippocampus between MRI (**B**), cell density (**C**), axon staining (**D**), astrocytes (**E**), microglia (**F**) and blood vessels (**G**). Similar alignment of ODFs was seen in the white matter (WM) when dMRI was compared with astrocyte and axon staining, while alignment in the stratum radiatum (SR) was similar comparing dMRI, astrocytes and blood vessels.

In order to determine a histological correlate for the orientational data that can be calculated from dMRI using constrained spherical deconvolution (CSD)^26^, structure tensor analysis was performed on the 3D histological data sets. Distinct orientation density functions (ODFs) were observed with MRI and histological tissue imaging, which could be associated with different layers of the hippocampus and surrounding tissue. In the white matter of the corpus callosum there was a clear left-right alignment of ODFs in dMRI maps along the length of the corpus callosum (Fig. 6A,B). An equivalent alignment of the ODFs was observed with the structure tensor analysis of the neurofilament axon staining (Fig. 6D), GFAP-positive astrocytes (Fig. 6E) and, to a lesser extent, the lectin-stained blood vessels (Fig. 6G). The pyramidal layer of the hippocampus (Fig. 6B) has a largely isotropic orientation from dMRI maps, which was consistent with the structural tensor analysis in all cellular populations. However, in some voxels the dMRI ODFs can be seen to be influenced by the surrounding tissue, a partial volume contamination likely to be a function of the imaging resolution. In the stratum radiatum there was a distinct radial alignment of the ODFs in the dMRI, which was also reflected in the histological staining of axons, astrocytes and blood vessels (Fig. 4D,E,G). Medial-lateral alignment of fibres in the polymorphic layer (hilus) of the dentate gyrus could also be recognised in the structure tensor analysis of neurofilament staining, but was not visible with dMRI ODFs. The microstructure shown by Iba1 microglia staining and DAPI stained cell nuclei was predominately isotropic in all layers of the hippocampus.

## Discussion

By performing voxel-wise analysis of histological data, registered to MR images using our 3D-BOND pipeline, we have been able to show local differences in diffusion characteristics that correlate with a number of cellular components of the brain microstructure.

For voxel-wise analysis of diffusion MR and histological data, it is necessary to broaden the coverage of histological analysis and increase the resolution of the MRI. *Ex vivo* MR imaging allows longer scan times and higher resolution brain imaging of sufficient signal-to-noise to facilitate 3D landmark registration and subregion analysis of brain microstructure, and was therefore utilised in this study. *Ex vivo* MRI has the potential limitation of altered tissue structure and water diffusion due to the fixation process (or tissue degradation in the case of sub-standard fixation). However, recent work from Dyrby and colleagues shows that appropriate fixation protocols and rehydration procedures, such as those used here, allow stable, biologically relevant dMRI to be performed *ex vivo*^27^. Minor modifications were made from the study of Dyrby and colleagues, to adjust for the different size of the mouse brains (compared with pig), and processing times were keep consistent to allow comparison between samples, as fixation time and time in PBS has clearly been shown to affect tissue MRI signal and tissue size (de Guzman *et al*. 2016). Wu & Zhang^28^ have shown that good concordance is possible between high resolution *in vivo* and *ex vivo* dMRI within the hippocampus of mice. In choosing the resolution for diffusion MRI in this study, we aimed to use an imaging resolution that would facilitate i) delineation of tissue subregions, ii) registration to high resolution histology and iii) meaningful comparison with the *in vivo* and *ex vivo* imaging currently used in the research field. In future, it will be necessary to compare multiple imaging parameters to determine how resolution affects the associations with microstructure (e.g. through partial volume effect), and therefore how generalizable are the relationships between histological features and diffusion metrics. The advanced imaging paradigms currently being prepared for both *in viv*o and *ex vivo* imaging^28–30^, will be advantageous here. However, we also need to ensure that the lower resolution imaging possible in the majority of pre-clinical animal studies^5,31,32^ can be interpreted in the light of this work.

We chose the CLARITY technique^13^ for 3D microstructural light imaging of the brain as it is compatible with immunohistochemistry, unlike methods such as SeeDB^12^, Sca*l*e^11^ or modified-BABB^9^. The hydrogel monomer used in the CLARITY protocol facilitates the maintenance of tissue structure and integrity through this clearing process. Cellular structure is consistent with standard immunohistochemistry and a variety of antibodies prove useful for the detection of distinct cellular populations. Tissue deformation due to shrinkage and swelling is part of all histological tissue processing methods; in order to minimise both protein loss and tissue deformation with the CLARITY method we utilised a passive clearing process^21^. We report an RMS distance between MRI and CLARITY landmarks of 0.17 ± 0.03 mm, an error that reflects both minor deformation of the CLARITY-processed tissue and accuracy of both the landmark placement and registration. This error is lower than previous values of 0.26 ± 0.14 mm^28^ and 0.59 ± 0.64 mm^33^ reported in other studies registering MRI to histological data. Selection of voxels for analysis from medial areas of the hippocampus minimised the effective RMS, as the registration error was greatest on the lateral margins.

Fractional anisotropy and mean diffusivity can be seen to differ throughout the hippocampus, with regional differences in radial and parallel diffusivity largely similar to mean diffusivity in this brain structure. Diffusivity is higher in DGgl than the CA1sp and DGml of the hippocampus, and correlates with cell and axonal density, and to a lesser degree blood vessel content of voxels. A regional variation in mean apparent fibre density was also observed throughout the hippocampus that differed in pattern to the DTI metrics. This measure of tissue microstructure correlated with axon density, and to a lesser extent voxel astrocyte and blood vessel content.

The positive correlation between mean diffusivity and cell density was not expected given current microstructural models. However, a high apparent diffusion coefficient (ADC) has previously been reported in the CA1sp and DGgl of the hippocampus and it has been shown to decrease with cellular loss due to injury^34^. There are a number of possible interpretations for this data, which need to be specifically studied in the future, however, the reproducibility of this finding across a number of registered voxels and in multiple tissue samples suggests positive correlation can exist between mean diffusivity and cell density in some brain regions.

When comparing the CA1sp and DGgl for secondary factors that may modulate diffusivity within these areas, a correlation between cell density was still observed. The size of the cells and their apparent density, as shown with DAPI staining, within the CA1sp region and the DGgl are very different, explaining the strength of this correlation. This finding needs to be further explored with additional b-values and microstructural features, and local and general patterns of microstructure and diffusivity will need to be determined. Our bespoke pipeline 3D-BOND will prove invaluable for this assessment, enabling accurate correlation of MR images acquired with an extended range of b-values and more complex microstructural analysis performed over a wider range of brain regions.

The highest correlation between diffusivity and tissue microstructure was found for mean and radial diffusivity and mean apparent fibre density, correlating with the axonal content in the voxel. This is also a counter-intuitive correlation, as usually areas with high axonal density (e.g. white matter tracts) are associated with low mean diffusivity and high apparent fibre density. It is not clear at this stage what may be driving this correlation, although it may be related to the fact that axonal density is relatively low in the regions of the hippocampus studied here. Of note, the positive correlation between mean diffusivity and neurofilament staining is not just a function of cell density within the hippocampal layers, as DAPI and NF do no correlate directly on a voxel-by-voxel basis. Possibly of greater importance is the correlation between dMRI ODFs and those calculated from structure tensor analysis of the neurofilament staining. Alignment between diffusion tensors and structure tensors has previously been shown in a small region of the human hippocampus^35^. In this study, there is clear alignment between orientation of axons and dMRI ODFs in the white matter and stratum radiatum, highlighting the sensitivity of such methods to variations in microstructural tissue organisation, and specifically to neurofilament arrangement. This suggests that such metrics may prove informative to studies of developmental or pathological variation in brain microstructure over other, rotationally-invariant, diffusion metrics.

Blood vessels are typically considered to be evenly distributed through the brain and to have flow in multiple directions within each voxel at the microstructural level; additionally the signal from large vessels is attenuated at very low b-values (~20 s/mm^2^ ^36^). As a result, the contribution of water movement in blood vessels is not generally considered in dMRI models^36,37^. The negative correlation between diffusivity and vascular density (~R^2^ = 0.19) did not retain statistical significance following FDR comparison. Further work will be required to determine if this lack of statistical significant relationship is maintained at different resolutions, b-values or in perfused tissue. While some directionality was observed with structural tensor analysis of the vasculature network, it is likely that these conform to the underlying microstructural elements, such as axon pathways, rather than conferring significant orientation on the tissue in their own right^38^. Altered vascular density is observed in a number of neurological disorders, e.g. stroke, and 3D-BOND maybe useful in further exploring regional variation in normal vascular density, and the sensitivity of dMRI to local changes in the vasculature. In the case of the microvasculature, it will be necessary to also perform dMRI *in vivo* rather than just *ex vivo*, as patent, flowing vascular networks will have a different effect on diffusion signal at certain b-values^37^ than that suggested by the microstructure alone, and neuronal activity has been implicated in inducing diffusion changes where hemodynamic changes may be a contributing factor^39^.

Numerous studies have predicted that astrocytes can contribute to dMRI metrics^40–42^, a position supported by the voxel-wise data produced in this study. Fractional anisotropy positively correlated with GFAP density across the hippocampus, a measure that included both the cell body and processes of the astrocytes. Interestingly, the CA1sr, which has the highest fractional anisotropy value measured in this study, also shows a clear radial alignment of the tissue that can be identified in ODF maps of the dMRI and astrocyte staining, with astrocyte processes contributing to the radial alignment (as well as axon alignment). Astrocytes proliferate, change morphology and GFAP production with injury, and it is likely that for this cellular population (and many of the others discussed here), it is not just the density of cells within a voxel that is important, but also the orientation of major cellular structures. Budde *et al*.^40^, showed the significant contribution of astrocytes to tensor-based tractography following closed-cortical injury in mice using structure tensor analysis of 2D-histology, highlighting the importance of considering alignment of the cellular microstructure in addition to the overall tissue content. The capacity to perform structure tensor analysis in 3D, which is facilitated by the 3D-BOND pipeline, will be key to advances in this area. In the hippocampus and white matter the majority of astrocytes express GFAP at rest, other markers that show both the astrocyte cell body and processes, such as Glutamine synthase and ALDH1L1, will need to be utilised in other brain regions.

### Generalisability of hippocampal diffusion and limitations of this study

While this study provides evidence of association between diffusion metrics in the hippocampus and specific cell populations, it is important to note that these are not necessarily generalizable to the *in vivo* brain; the local tissue features of the hippocampus are sufficiently different from other brain regions to suggest that these findings should not be directly applied to other tissue without further experimentation. Likewise, the correlations are likely to change with different resolution and b-value for dMRI. The acquisition parameters of the present study were chosen to be broadly consistent with the standard in the field of *ex vivo* mouse brain imaging, so that the data could be as meaningful to other researcher as possible. However, more work with this method and different acquisition parameters will be required before we have a clear conception of the biological correlates underpinning dMRI. In the meantime, 3D-BOND has provided significant information for the interpretation of hippocampal injury, where changes in astrocyte and neuronal density are commonly seen in disease^43^.

Bridging the gap in scale between macroscopic (e.g. MRI) and microscopic (histological) data through registration of large-scale 3D datasets, we present a robust technique for comparing neuroimaging modalities and tissue microstructure on a voxel-by-voxel basis. This method allows the validation of acquisition parameters (e.g. the relationship between b-values or diffusion times and tissue-dependent diffusion) or analytical models (e.g. DTI compared with multi-shell analysis models, such as NODDI^44^ or multi-tissue CSD^45^) or the sensitivity of novel imaging techniques, such as ultrasound-based super-resolution vascular imaging^46^. As well as validation of existing techniques, combined investigations in the future will allow development of disease-specific biomarkers, new acquisition parameters and analytical models. 3D-BOND can also be used in models of brain injury and development to determine the specificity and sensitivity of neuroimaging metrics to changes within the brain. We have already shown correlations between mean diffusivity and a number of cellular elements within the hippocampus, as well as orientation alignment between diffusion ODFs and cell-specific microstructure. 3D-BOND has the potential to measure injury and treatment effect sizes detectable with dMRI and other neuroimaging techniques and to inform the search for biomarkers of clinical assessment and neuroprotective drug trials.

## Electronic supplementary material

Supplementary information

Supplementary Figure 1

Supplementary Figure 3
