# Supplementary material for: Voxel-wise comparisons of cellular microstructure and diffusion-MRI in mouse hippocampus using 3D Bridging of Optically-clear histology with Neuroimaging Data (3D-BOND)
Source: Sci Rep. 2018 Mar 5;8:4011. doi: 10.1038/s41598-018-22295-9 (PMC5838167; doi:10.1038/s41598-018-22295-9)
Supplement: Supplementary file 1 — Supplementary information [file 41598_2018_22295_MOESM1_ESM.pdf]

Voxel-wise comparisons of cellular microstructure and diffusion-MRI in mouse hippocampus using 3D Bridging of Optically-clear histology with Neuroimaging Data (3D-BOND)

Stolp HB [1], Ball G [1,3], So P-W[2], Tournier, J-D[1], Jones M[1], Thornton C[1]*, Edwards AD[1]
[1]Centre for the Developing Brain, Division of Imaging Sciences and Biomedical Engineering, St Thomas' Hospital, King's College London, London SE1 7EH, United Kingdom
[2]Department of Neuroimaging, Maurice Wohl Clinical Neuroscience Institute, Institute of Psychiatry, Psychology and Neuroscience, King's College London, London SE5 9NU, United Kingdom
[3]Developmental Imaging, Clinical Sciences, Murdoch Children's Research Institute, Melbourne 3052, Australia

Author email addresses: helen.stolp@kcl.ac.uk; gareth.ball@mcri.edu.au; po-wah.so@kcl.ac.uk; jacques_donald.tournier@kcl.ac.uk, marc.jones@kcl.ac.uk; claire.thornton@kcl.ac.uk; ad.edwards@kcl.ac.uk;

Corresponding Author:
*C. Thornton
Centre the Developing Brain
1st Floor, South Wing, St Thomas' Hospital
London SE1 7EH, United Kingdom
Email: claire.thornton@kcl.ac.uk
Phone: +44 (0) 20 7188 7118 x54387

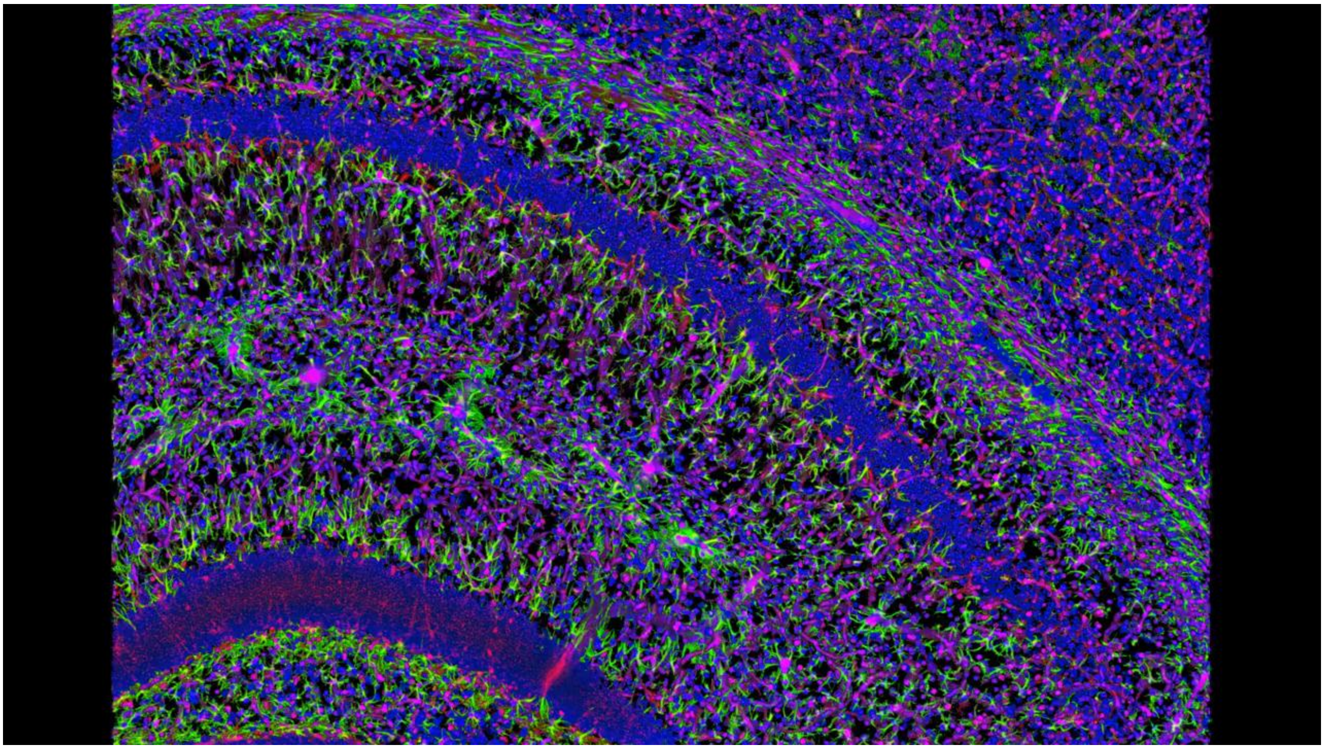

Suppl. Figure 1. 3D representation of microstructure in the hippocampus. Movie of GFAP-positive astrocytes (green), lectin-positive blood vessels (pink) and DAPI-positive cell nuclei (blue) in the hippocampus at a 1μm^3 voxel resolution.

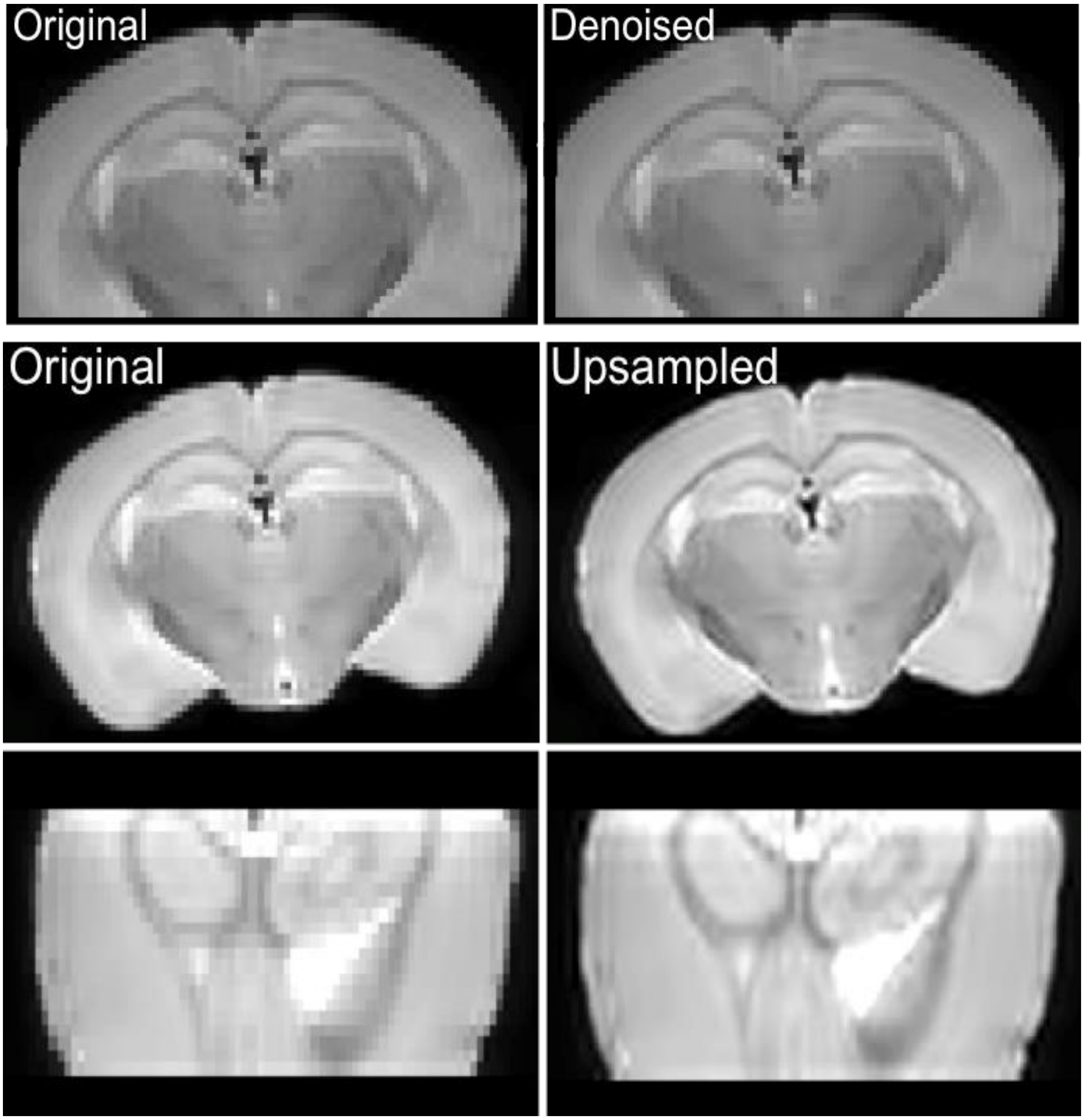

Suppl. Figure 2. MRI processing. For registration, averaged B0 images were denoised and upsampled to improve landmark identification.

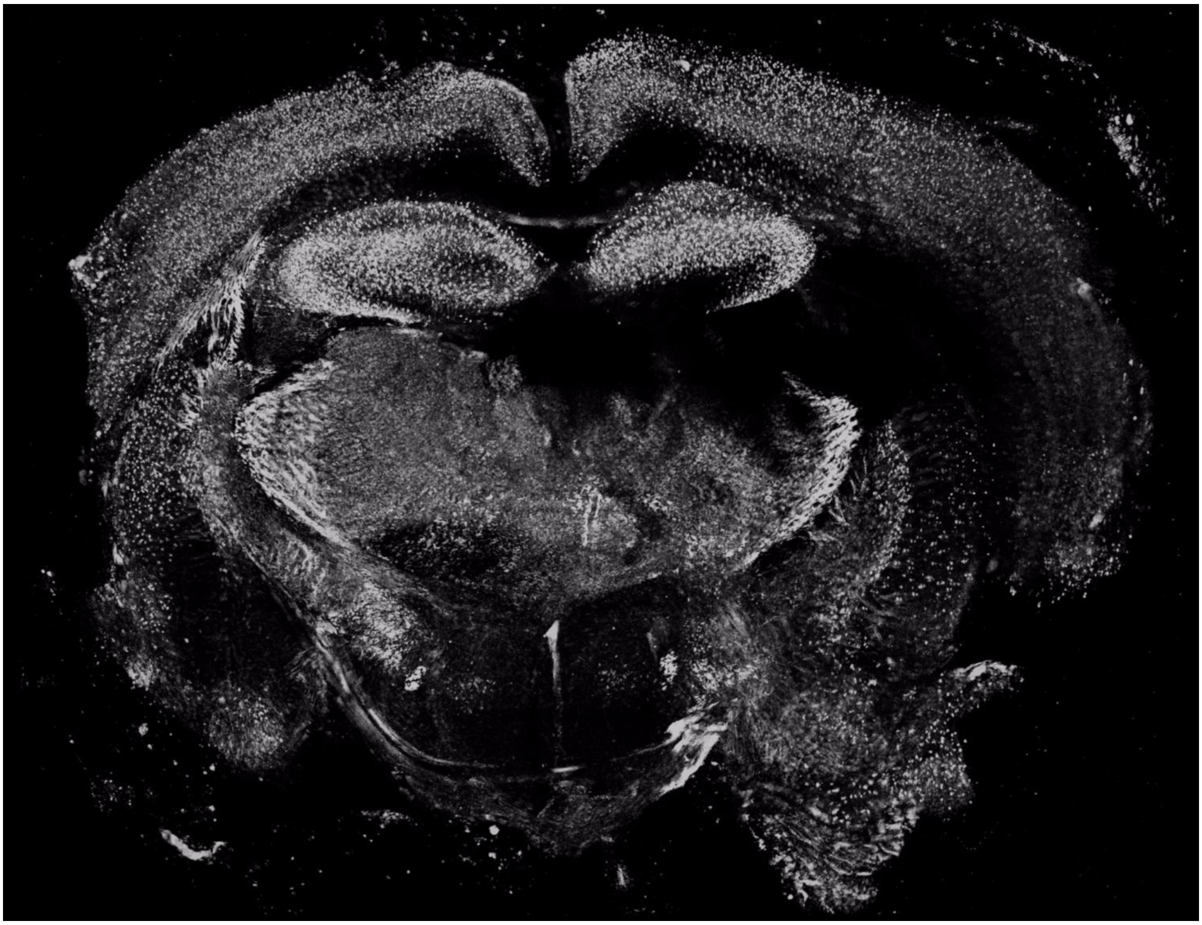

Suppl. Figure 3. 3D hippocampal structure*.* 3D projection of parvalbumin-positive interneurons in a 0.6mm tissue slice, from optically-clear tissue; the 3D structure of the hippocampus is clearly demonstrated. Scale bar = 1mm.

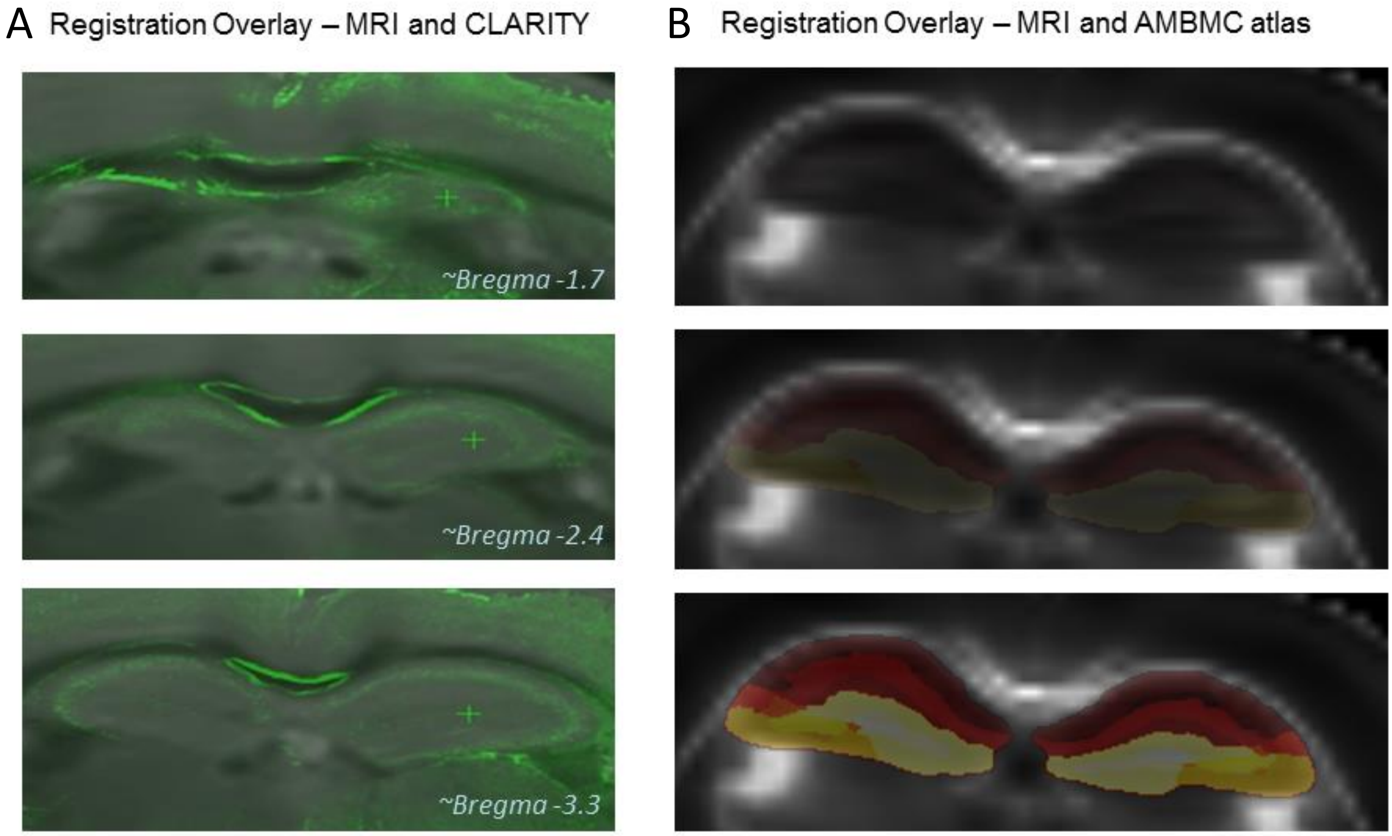

Suppl. Figure 4. Image processing and registration. A) Overlay of registered MRI (grey) and confocal (green) images at a number of rostro-caudal positions shows registration of the hippocampus. B) Overlay of hippocampal subregion mask from AMBMC atlas (colour) on FA map (grey), with varying transparency of the overlay to show the registration alignment between the two datasets.

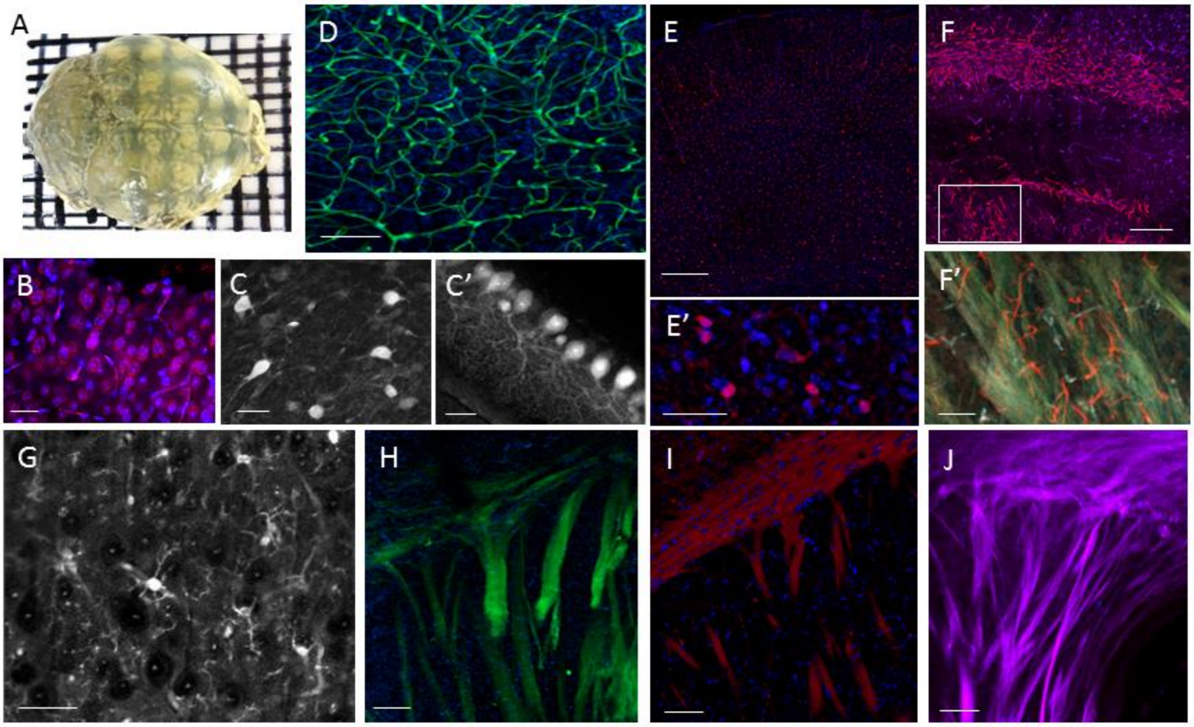

Suppl. Figure 5. Immunohistochemical visualisation of cellular microstructure in optically-clear tissue. Brain tissue was processed until optically-clear (A) and stained using modified immunohistochemistry. Neurons are recognised with NeuN (B) or the interneuron marker, parvalbumin (C - cortex, C’ - cerebellum). Blood vessels can be detected using biotinylated Tomato lectin (D). Astrocytes can be identified throughout the brain using S100b (depicted in the cortex in E, higher magnification in E’), or in the white matter tracts and in association with blood vessels using GFAP (F and F’). Microglia stained with Iba1 (G). White matter tracts can be identified with myelin markers (PLP and CNPase respectively, H, I), or neurofilament (J). Scale bar: E, F = 200µm; D, H, I, J = 100µm; B, C, C’, E’, F’, G = 50µm.

Supplementary Table 1. Registration landmarks

Landmark no.	lateralisation	Bregma co-ordinate	Region description	Graphical representation
1	L &/or R	-1.80mm	Centre of the CA3 region of the hippocampus, when only the very first portion of the hippocampus and dentate gyrus are visible	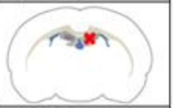
2	Midline	-1.80mm	Border between dentate gyri and third ventricle in same section	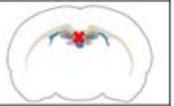
3	L &/or R	-3.30mm	Dorsal border of CA1 hippocampus, directly under peak of cingulum	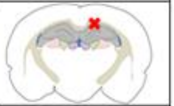
4	L &/or R	-3.30mm	Dorsolateral border of hippocampus near external capsule, at CA2	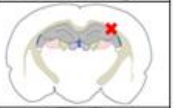
5	L &/or R	-3.30mm	Lateral boundary of CA2/3 region of the hippocampus, the fimbria hippocampus and lateral ventricle	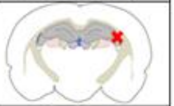
6	L &/or R	-3.30mm	Medial boundary of fimbria hippocampus where it joins the CA3 region of the hippocampus, near the lateral dorsal thalamic nucleus	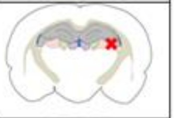
7	L &/or R	-3.30mm	Sulci between CA3 and blade of DG, between lateral posterior and lateral dorsal thalamic nuclei	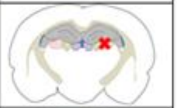
8	L &/or R	-3.30mm	Point of DG, between lateral posterior thalamic nucleus and habenular nucleus	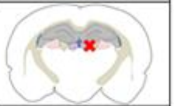
9	Midline	-3.30mm	Hippocampal bridge between hemispheres at top of third ventricle	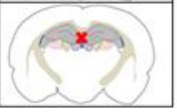
10	L &/or R	-3.30mm	Middle of hippocampus, on the hippocampal fissure immediately above the peak of the DG.	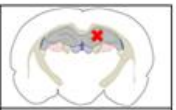
11	L &/or R	-4.30mm	Dorsal border of CA1 hippocampus, directly under peak of cingulum	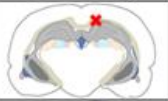
12	L &/or R	-4.30mm	Dorsolateral border of hippocampus near external capsule, at CA2	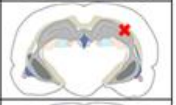
13	L &/or R	-4.30mm	Mid-point of fimbria hippocampus, between dorsal and ventral CA3 hippocampal region	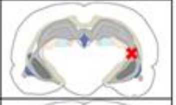
14	L &/or R	-4.30mm	Dorsal endopiriform nucleus, at base of external capsule	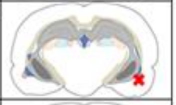
15	L &/or R	-4.30mm	Optic tract at lateral flexion of thalamus	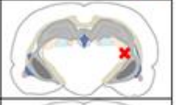
16	L &/or R	-4.30mm	Sulci between CA3 and blade of DG, between DLG and lateral posterior thalamic nucleus	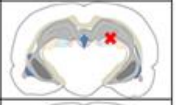
17	L &/or R	-4.30mm	Sulci between CA1 and DG, above pretectal nucleus	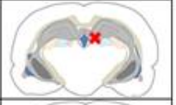
18	Midline	-4.30mm	Hippocampal bridge between hemispheres at top of third ventricle	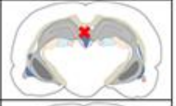
19	L &/or R	-4.30mm	Middle of hippocampus, on th hippocampal fissure immediately above the peak o the DG.	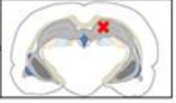

Supplementary Table 2. Details of registered tissue samples

Sample number	Position (relative to Bregma)	Landmarks (number)	Root mean square (RMS) distance (mm)
1, slice 1	-2.1mm to -2.8mm	37	0.134
1, slice 2	-2.8mm to -3.6mm	30	0.174
2, slice 1	-1.6mm to -2.9mm	32	0.196
			0.168 ± 0.031

Supplementary Table 3. Antibodies tested in CLARITY-processed tissue

Antibody/stain	Species	Cell /structure recognised	Dilution	Supplier and catalogue number		Staining success
NeuN	Guinea Pig	Neuron	1:400	Millipore	ABN90P	Yes
MAP1B	Mouse	Neuron	1:200	Abcam	ab3095	No
MAP2	Rabbit	Neuron	1:200	Abcam	ab32454	No
Parvalbumin	Rabbit	Interneurons	1:400	Abcam	ab11427	Yes
Calretinin	Mouse	Interneurons	1:100	Millipore	MAB1568	No
GAD_{67}	Mouse	interneurons	1:100	Millipore	MAB5406	No
Iba1	Rabbit	Microglia	1:200	Wako	019-19741	Yes
Cx3cr1	Rabbit	Microglia	1:200	Abcam	ab8021	No
F4/80	Rat	Microglia	1:200	Abcam	ab6640	No
Tomato lectin	N/A	Blood vessels	1:400	Vector	B-1175	Yes
GFAP	Goat	Astrocytes	1:400	Abcam	ab53554	Yes
S100b	Rabbit	Astrocytes	1:400	DAKO	Z0311	Yes
Glutamine Synthetase	Rabbit	Astrocytes	1:200	Sigma	G2781	No
Olig2	Rabbit	Oligodendrocytes	1:200	Millipore	AB9610	Yes
PLP	Mouse	Myelin	1:100	Millipore	MAB388	Yes
CNPase	Mouse	Myelin	1:100	NeoMarkers	MS_349_P	Yes
Neurofilament	Chicken	Axons	1:400	Millipore	AB5539	Yes
NeuroTrace	N/A	Neuron	1:50	Molecular Probes	N21482	Yes

Supplementary results

In CLARITY-processed, optically cleared tissue (Suppl. Figure 5A), immunohistochemistry was used to detect a number of cellular populations. Neurons could be distinctly recognised with NeuN antibody (Suppl. Figure 5B) and NeuroTrace561 dye, but not with microtubule associated protein (MAP)1b and MAP2. Interneuron populations could be robustly detected with anti-parvalbumin antibodies (Suppl. Figure 5C, 5C'), but not with calretinin or glutamate decarboxylase 1 (GAD_{67}). Tomato lectin delineated blood vessels in CLARITY-treated tissue (Suppl. Figure 5D), but did not recognise microglia. Cortical and white matter astrocytes could be identified with S100b (though typically with less than 200μm penetration depth in the CLARITY-treated tissue, Suppl. Figure 5E, 5E'), but not antibodies against glutamine synthetase. Glial fibrillary acid protein (GFAP) consistently stained fibrous astrocytes within the white matter and hippocampus (Suppl. Figure 5F, 5F'). Resting and activated microglia were visible ubiquitously through the brain with an antibody against Iba1 (Suppl. Figure 5G), but not F4/80, Cx3cr1 or CD11b. The cell bodies of oligodendrocytes were identified by the oligodendrocyte transcription factor, Olig2 (data not shown), while myelinated fibres were stained with antibodies against proteolipid protein (PLP, Suppl. Figure 5H) and 2',3'-cyclic-nucleotide 3'-phosphodiesterase (CNPase; Suppl. Figure 5I). Axons within the white matter tracts were recognised by the cytoskeletal protein, neurofilament (NF, Suppl. Figure 5J).
